# Methodological study to evaluate the psychometric properties of FACIT-CD in a sample of Brazilian women with cervical intraepithelial neoplasia

**DOI:** 10.1186/s12885-017-3676-8

**Published:** 2017-10-16

**Authors:** Cristiane Menezes Sirna Fregnani, José Humberto Tavares Guerreiro Fregnani, Adhemar Longatto-Filho

**Affiliations:** 1 Teaching and Research Institute of Barretos Cancer Hospital, Antenor Duarte Villela street, 1331. Barretos, São Paulo, Zip code: 14784-400 Brazil; 2 Life and Health Sciences Research Institute (ICVS), School of Health Sciences, University of Minho, 4710-057 Braga, Portugal; 3 ICVS/3B’s, PT Government Associate Laboratory, Braga, Guimarães Portugal; 4 Laboratory of Medical Investigation (LIM) 14, FMUSP, São Paulo, Brazil

**Keywords:** Cervical intraepithelial neoplasia, FACIT-CD, Psychometric properties, Human papilloma virus

## Abstract

**Background:**

The occurrence of cervical intraepithelial neoplasia (CIN) is associated with changes in health-related quality of life, including psychological factors, such as fear and shame, and changes in sexuality and sexual satisfaction, such as decreased sexual desire and frequency of sexual intercourse. Personal relationships are the most affected because CIN is sexually transmitted and many women tend to blame their partner for disease transmission. The aim of this study was to evaluate the psychometric properties of the FACIT-CD questionnaire in Brazilian women diagnosed with CIN.

**Methods:**

The properties of the FACIT-CD questionnaire were tested on a sample of 439 women seen at the Department of Prevention of Barretos Cancer Hospital, including 329 patients who were diagnosed with CIN and 110 women who were not diagnosed with the disease. The analysed parameters included internal consistency (Cronbach’s alpha), reproducibility (intraclass correlation coefficient), structural validity, convergent validity (correlation with the SF-12 and EORTC QLQ-CX24 questionnaires), discriminant validity (according to disease status, and self-rating of health), sensitivity, and responsiveness.

**Results:**

The Cronbach alpha values ​​of the FACIT-CD scales were higher than 0.70 with the exception of the relationship scale (0.66). The FACIT-CD reproducibility was satisfactory, with variation in the intraclass correlation coefficients ranging between 0.50 and 0.83, although the 95% confidence interval (CI) was lower than 0.40 (0.33–0.64) on the treatment satisfaction scale. Regarding structural validity, only one item on the physical well-being scale was not kept in the original domain. The expected correlations between the FACIT-CD and SF-12 were not confirmed, whereas the correlations between the FACIT-CD and EORTC QLQ-CX24 were confirmed. The questionnaire was able to discriminate the groups according to disease status and self-rating of health. The sensitivity was low for the relationship scale and moderate for the other scales. The responsiveness of the FACIT-CD questionnaire varied between the groups that denominate the self-perception of health as no change, improvement or worsening.

**Conclusion:**

Our results are encouraging and indicate that the FACIT-CD questionnaire is a promising tool for the analysis of the quality of life of women with CIN.

## Background

Human papillomavirus (HPV) infection is the most prevalent sexually transmitted disease worldwide [1]. Approximately 440 million people are estimated to have genital HPV infections worldwide [2], and approximately 10% of women will carry HPV at some point in their life [3].

Approximately 40 types of HPV can invade the mucous membranes of the upper aerodigestive tract and anogenital region of humans; these HPV types are classified as low and high risk according to their carcinogenic potential [4]. Low-grade intraepithelial lesions spontaneously regress in 60% of cases, and only 10% of cases progress to high-grade lesions. Even cervical carcinoma in situ (CIN 3) may undergo spontaneous regression to normality in one-third of women [4]. The period from HPV infection to the onset of invasive cervical cancer is estimated to extend 10 to 20 years, which makes this disease preventable using well-structured screening strategies [5].

The occurrence of cervical intraepithelial neoplasia is associated with changes in health-related quality of life (HRQoL), including psychological factors, such as fear and shame, and changes in sexuality and sexual satisfaction, such as decreased sexual desire and decreased frequency of sexual intercourse [6–8]. Such problems tend to sustain for a period of time after the treatment [9]. Anxiety, distress, concern with fertility, changes in family dynamics and work-related changes are also negative effects of CIN diagnosis and treatment [10–13]. Because this disease is sexually transmitted, many women tend to blame their partner for transmission [13, 14].

Despite the availability of instruments to objectively assess HRQoL, few instruments have investigated the impact of HPV infection in the female genital tract. The number of studies on aspects related to HRQoL in women diagnosed with cervical cancer has significantly increased. This increased interest can be justified by the magnitude of the disease, which predominantly affects young women who will live the rest of their lives with the consequences of the disease and treatment [15–18]. However, little is known about the impact of diagnosis and treatment on HRQoL in women diagnosed with precursor lesions of cervical cancer.

In 2010, Rao et al. [6] developed a tool that was designated the Functional Assessment of Chronic Illness Therapy – Cervical Dysplasia (FACIT-CD) to assess the functional, physical, and psychological characteristics of women with CIN. The questionnaire has recently been translated and adapted to Brazilian Portuguese.

The FACIT system questionnaires are easy to apply (self-applied or using interviews), require little time to complete, have adequate validity and sensitivity to detect changes, and are designed to reach a population with a level of education corresponding to the fourth year of primary school (9–10-year-old age group) [19].

The aim of this study was to evaluate the psychometric properties of the FACIT-CD questionnaire in Brazilian women diagnosed with CIN.

## Methods

This methodological longitudinal study was conducted in the Department of Prevention and Oncological Gynaecology of the Barretos Cancer Hospital, Barretos, state of São Paulo, Brazil. A total of 439 women were eligible, including 329 women with a histopathological diagnosis of CIN (low or high grade) without treatment and 110 women not diagnosed with the disease. The participants attended the Department of Prevention for screening via a cervical cytology examination (Papanicolaou test). Illiterate women and women known to have psychological or psychiatric disorders that could hinder the understanding of the questionnaire and the informed consent form were excluded.

After formal agreement to participate in the study, the participants answered the questionnaires, which were applied using interviews by a single interviewer. Sociodemographic and clinical data were initially collected. Then, the FACIT-CD, EORTC QLQ-CX24, and SF-12 (version 2) questionnaires were applied; this step was considered the first stage of the study.

Among the 329 women diagnosed with CIN, the first 112 were selected to answer the FACIT-CD questionnaire a second time to assess the reproducibility of the instrument. Interviews were conducted in a second consultation 30 days after the first interview to inform the test results. Of the 112 women selected, 87 (77.7%) returned on the expected date and answered the questionnaire.

The responsiveness and sensitivity of the FACIT-CD questionnaire were evaluated in 228 participants with a medical indication for surgical treatment using the loop electrosurgical excisional procedure (LEEP). Of this total, 179 (78.5%) returned after treatment during the stipulated period (4–6 months) and answered the FACIT-CD questionnaire a second time and the first question of the SF-12 questionnaire (“In general, would you say your health is:”). The responses obtained to this question at baseline and after treatment allowed the creation of groups and the classification of women as having improved health, worsened health, or no change in health. Among the other participants who underwent LEEP (49 women), 7 presented with invasive carcinoma and were forwarded to the Department of Oncological Gynaecology, 12 women returned outside the period stipulated for re-application of the questionnaire, and the remaining participants did not return on the previously scheduled date.

### FACIT-CD questionnaire

The FACIT-CD instrument in Brazilian Portuguese is a specific instrument to assess the HRQoL of women with CIN and comprises 37 questions divided into five scales to assess aspects related to physical well-being (9 questions), treatment satisfaction (4 questions), general perception (7 questions), emotional well-being (11 questions), and relationships (6 questions). The scores were calculated using the specific guidelines provided by the FACIT [20]. The responses were based on experiences from the last 7 days. The answer scale is Likert, with scores ranging between 0 and 4 (a little bit to very much). A score was assigned to each scale, and the scores were summed to obtain a single value. The total score of the questionnaire ranged from 0 to 136. A higher score indicated a better HRQoL.

### EORTC QLQ-CX24 questionnaire

The EORTC QLQ-CX24 questionnaire was developed and validated cross-culturally by the European Organization for Research and Treatment of Cancer and was used for the assessment of HRQoL in patients with cervical cancer [21]. This instrument consists of 24 questions divided into three scales of multiple items and six scales of single items, including 11 questions on symptoms (questions 31 to 37, 39, and 41 to 43), 3 questions on body image (questions 45 to 47), 4 questions on sexual/vaginal function (questions 50 to 53), 1 question on lymphedema (question 38), 1 question on peripheral neuropathy (question 40), 1 question on menopause symptoms (question 44), 1 question on sexual worry (question 48), 1 question on sexual activity (question 49), and 1 question on sexual enjoyment (question 54). The scores were calculated separately for each scale of the multiple and single items to allow the evaluation of sexuality using the questions on sexual/vaginal function, sexual activity, and sexual enjoyment [21].

### SF-12 questionnaire

The SF-12 questionnaire is a generic instrument for the assessment of HRQoL. This questionnaire is considered a smaller version of the Medical Outcomes Study 36 – Item Short-Form Health Survey (SF-36). The main goal of developing an instrument with a reduced number of items was to provide a questionnaire that could be answered quickly and easily, which is a good option for population-based studies and health screening [22]. The questionnaire consists of 12 questions derived from the SF-36 questionnaire. In Brazil, the SF-36 questionnaire was translated into Brazilian Portuguese and validated by Ciconelli et al. in 1999 [23]. The scores were calculated using specific software provided by the Medical Outcomes Health Survey.

### Analysis of psychometric properties

The classical psychometric properties of the FACIT-CD questionnaire were tested by assessing the internal consistency, reproducibility, structural validity, convergent and divergent validity, known-group validity, sensitivity, and responsiveness.

Cronbach’s alpha coefficient was used to test the internal consistency of the instrument, with values equal to or higher than 0.70 considered appropriate [24]. The reproducibility of the FACIT-CD was evaluated by comparing the scores obtained in the questionnaire during the first and second interviews. For this purpose, the intraclass correlation coefficient (ICC) was used. Structural validity was assessed using a confirmatory factor analysis. The oblique rotation method was used for principal component analysis, and a five-factor solution was forced, as presented in the original questionnaire. For the analysis of convergent and divergent validity, the scores generated by the FACIT-CD questionnaire were correlated with the scores generated by the SF-12 questionnaire and the scores of the scales that assessed sexuality in the EORTC QLQ-CX24 questionnaire. The Spearman correlation coefficient was used to calculate the correlations, with values ​higher than 0.40 considered appropriate [25]. The assumptions of correlations between the FACIT-CD, SF-12, and EORTC QLQ-CX24 scales were established a priori.

To assess the known-group validity, women without the disease were compared with women diagnosed with CIN using the Mann-Whitney test. These two groups were also assessed based on the answers to the first question of the SF-12 (“In general, would you say your health is:”). The responses were classified as excellent/very good, good, and poor/very poor and were compared using the Kruskal-Wallis test.

Sensitivity was evaluated by calculating the magnitude of the effect using the Cohen’s D, standardized response mean (SRM), and relative efficiency tests [26]. The tests were applied to the groups before and after treatment.

Responsiveness was analysed using hypotheses established a priori. For this purpose, the study groups were compared before and after treatment (LEEP). The reference statistical method most commonly used to measure the magnitude of changes in HRQoL scores is the assessment of the effect size (ES) and the SRM [27, 28], which provide useful data concerning significant changes in clinical practice [29]. The ES and SRM are defined using Cohen’s criteria, in which values ​​up to 0.20 indicate low responsiveness, values up to 0.50 indicate moderate responsiveness, and values higher than 0.80 indicate high responsiveness [26, 30]. The level of significance was 5% in all statistical tests.

### Ethical considerations

This study was approved by the Research Ethics Committee of the Barretos Cancer Hospital under CAAE No. 36619714.9.0000.5432, and all the women who agreed to participate in the study signed an informed consent form.

## Results

The characteristics of the study sample are shown in Table 1. The mean age of the women was 35.2 ± 10.1 years; most participants had a low education level and were Caucasian, married, and worked from home. The most common cytological result was a high-grade squamous intraepithelial lesion (ASC-H), and the most common histopathological result was CIN 2/3.

**Table 1:** Sociodemographic and clinical characteristics of the study sample

Variable	Description	Diagnosed with CIN	Not diagnosed with CIN
		*N* = 329	*N* = 110
Age	(Mean age)	35.2	48.5
Years of study	≤ 8 years	175 (53.2%)	58 (52.7%)
	> 8 years	154 (46.8%)	52 (47.3%)
Race	Caucasian	239 (72.6%)	82 (74.6%)
	Mixed	46 (14%)	13 (11.8%)
	Black	42 (12.8%)	11 (10%)
	Asian	2 (0.6%)	4 (3.6%)
Marital status	Married/cohabitating	175 (53.2%)	77 (70%)
	Single	103 (31.3%)	13 (11.8%)
	Separated/Divorced	37 (11.2%)	14 (12.7%)
	Widow	14 (4.3%)	6 (5.5%)
Occupation	Works from home	87 (26.4%)	4 (31%)
	Housewife	43 (13.1%)	16 (14.4%)
	Rural worker	17 (5.2%)	2 (1.8%)
	Saleswoman	17 (5.2%)	2 (1.8%)
	Other	165 (50.1%)	56 (51%)
Cytological result	NILM	12 (3.6%)	106 (96.4%)
	ASCUS	23 (7%)	4 (3.6%)
	ASCH	148 (45%)	–
	AGC	5 (1.5%)	–
	LSIL	54 (16.4%)	–
	HSIL	87 (26.5%)	–
Histological results	CIN I	133 (40.4%)	–
	CIN II/III	195 (59.3%)	–
	Invasive cancer	1 (0.3%)	–

*NILM* Negative for intraepithelial lesion or malignancy, *ASCUS* Atypical squamous cells of undetermined significance, *ASCH* Atypical squamous cells – cannot exclude HSIL, *AGC* Atypical Glandular Cells not otherwise specified, *LSIL* Low grade squamous intraepithelial lesion, *HSIL* High grade squamous intraepithelial lesion, *CIN* cervical intraepithelial neoplasia

Table 2 shows the descriptive statistical analysis conducted using the scores obtained in each of the scales and the corresponding Cronbach’s alpha coefficients and intraclass correlation coefficients (ICC). Only the relationship scale presented a Cronbach’s alpha coefficient smaller than 0.70, with a value of 0.66. The coefficients that evaluated the reproducibility of the FACIT-CD questionnaire scales ranged between 0.50 and 0.83; however, the lower limit of the 95% CI was smaller than 0.40 only on the treatment satisfaction scale.

**Table 2:** Cronbach’s alpha coefficients and intraclass correlation coefficients of the FACIT-CD questionnaire

Scale	Mean (SD)	Median	Minimum-maximum	Variation	Cronbach’s alpha	Intraclass correlation coefficient (95% CI)
Physical well-being	23.4 (4.2)	24.0	9–28	0–32	0.70	0.74 (0.62–0.82)
Treatment satisfaction treatment	9.7 (1.9)	9.0	3–12	0–16	0.77	0.50 (0.33–0.64)
General perceptions	18.8 (3.8)	19.0	5–24	0–28	0.76	0.72 (0.51–0.84)
Emotional well-being	30.6 (7.0)	32.0	5–40	0–44	0.79	0.76 (0.65–0.84)
Relationships	8.6 (2.2)	9.0	1–12	0–16	0.66	0.67 (0.54–0.77)
FACIT-CD	91.1 (11.6)	92.0	59–115	0–136	0.73	0.83 (0.75–0.89)

*SD* Standard deviation, *CI* Confidence interval

In the known-group validity analysis, the comparison between the groups of women with and without a diagnosis of the disease indicated significant differences in the average scores on all FACIT-CD questionnaire scales. Considering the health status rating by each participant, the group of women who rated their health as excellent/very good had significantly higher scores on all scales compared with the groups that rated their health as good or fair/poor (Table 3).

**Table 3:** Known-group validity of the FACIT-CD questionnaire

Scale	Women diagnosed with CIN (*N* = 329)	Women not diagnosed with CIN (*N* = 110)	p*	Excellent/Very Good (*N* = 90)	Good (*N* = 147)	Regular/Poor (*N* = 92)	p**
	Mean (SD)	Mean (SD)		Mean (SD)	Mean (SD)	Mean (SD)	
Physical well-being	23.4 (4.2)	24.8 (3.8)	< 0.001	25.2 (8.66)	23.8 (3.9)	21.0 (4.8)	< 0.001
Treatment satisfaction	9.7 (1.9)	0.6 (0.6)	< 0.001	11.0 (9.63)	9.7 (1.7)	9.2 (1.6)	< 0.001
General perceptions	18.8 (3.8)	13.9 (2.6)	< 0.001	21.6 (8.71)	18.9 (3.3)	16.6 (4.3)	< 0.001
Emotional well-being	30.6 (7.0)	39.8 (0.4)	< 0.001	32.6 (9.72)	30.2 (7.0)	30.0 (7.3)	0.048
Relationships	8.6 (2.2)	2.8 (0.6)	< 0.001	10.4 (9.62)	8.4 (2.1)	8.0 (2.3)	< 0.001
FACIT-CD	91.1 (11.6)	81.7 (4.9)	< 0.001	96.4 (10.26)	91.1 (11.0)	85.8 (11.4)	< 0.001

*CIN* cervical intraepithelial neoplasia

*p = Mann-Whitney; p** = Kruskal-Wallis

Regarding the structural validity of the FACIT-CD questionnaire (Table 4), the factor components were similar to those of the original questionnaire. The only exception was question GP5 ("I am bothered by side effects of treatment"); although this question belonged to the physical well-being domain in the original questionnaire, it presented higher factor loading in the emotional well-being domain.

**Table 4:** Factor analysis of the FACIT-CD questionnaire (*N* = 329)

Scale	Item	Question	Component				
			1	2	3	4	5
Physical	CD1	I have discomfort in my pelvic area (lower part of the stomach)	−0.016	−0.025	0.703	0.011	0.011
well-being	CD2	I have pain in my pelvic area (lower part of the stomach)	−0.028	−0.098	0.701	0.023	0.100
	CD3	I have cramping in my pelvic area (lower part of the stomach)	0.027	0.052	0.572	0.003	0.004
	Cx1	I am bothered by discharge or bleeding from my vagina	0.213	0.221	0.496	−0.072	−0.177
	GP5	I am bothered by side effects of treatment	0.293	0.165	0.063	0.069	−0.180
	ES8	I have pain or discomfort with intercourse	0.065	−0.124	0.680	−0.090	0.118
	CD4	I have to limit my sexual activity because of the infection	0.122	−0.050	0.665	−0.049	0.014
	CD5	I worry about spreading the infection	0.390	0.097	0.403	0.080	−0.133
Treatment	GR1	I have confidence in my doctor	0.045	0.216	0.081	0.677	−0.012
satisfaction	CD6	I feel I have received the treatment that was right for me	−0.001	0.245	0.025	0.764	0.037
	CD7	My doctor gave me explanations that I could understand	−0.042	0.113	−0.115	0.775	0.201
	CD8	My doctor explained the possible benefits of my treatment	0.055	0.060	−0.111	0.768	0.079
General	GF1	I am able to work (including at home)	0.091	0.558	−0.012	0.159	0.195
perceptions	GF3	I am able to enjoy life	−0.129	0.768	0.014	0.083	0.136
	HI11	I am hopeful about the future	0.000	0.667	0.072	0.118	0.148
	Sp9	I find comfort in my faith or spiritual beliefs	0.013	0.613	0.059	0.100	0.128
	GF7	I am content with the quality of my life right now	−0.166	0.646	−0.252	0.001	0.116
	CD9	I feel that I can manage things that come up around this infection	−0.204	0.563	−0.111	0.255	−0.008
	CD10	I have accepted that I have this infection	−0.359	0.401	0.038	0.260	−0.037
Emotional	CD11	I worry that the infection will get worse	0.487	−0.056	0.274	0.022	0.012
well-being	CD12	I have hidden this problem so others will not notice	0.700	0.052	−0.050	0.046	−0.251
	CD13	I have concerns about my ability to become pregnant	0.354	0.022	0.065	0.108	0.209
	BMT18	The cost of my treatment is a burden on me and my family	0.389	−0.078	0.105	−0.046	0.240
	CD14	I worry about other people’s attitudes towards me	0.661	−0.223	0.037	0.044	−0.028
	CD15	I feel embarrassed about the infection	0.681	−0.163	0.145	−0.015	−0.057
	CD16	I tend to blame myself for the infection	0.565	−0.062	0.002	−0.028	−0.070
	CD17	I was careful who I told about the infection	0.434	0.214	0.011	0.106	−0.190
	CD18	I have had difficulty telling my partner/spouse about the infection	0.529	0.106	−0.052	−0.020	−0.155
	CD19	I am frustrated by the infection	0.743	−0.172	0.045	−0.069	0.030
	CD20	I am depressed about the infection	0.651	−0.324	0.061	−0.033	0.127
Relationships	CD21	I get emotional support from my partner/spouse	−0.072	0.147	−0.015	0.032	0.721
	CD22	I get emotional support from family members	−0.121	0.146	0.043	−0.019	0.722
	GS1	I feel close to my friends	−0.065	0.252	0.030	0.176	0.372
	HI3	I have people to help me if I need it	−0.023	0.302	0.018	0.185	0.630

The convergent analysis results of the FACIT-CD questionnaire are shown in Table 5. The correlation between the FACIT-CD and SF-12 scales was weak (r_s_ < 0.40). The correlation between the FACIT-CD and EORTC QLQ-CX24 scales was moderate (*r* = 0.40–0.60), which confirmed previously established assumptions.

**Table 5:** Correlation coefficients between the FACIT-CD, SF-12, and EORTC QLQ-CX24 questionnaire scales (convergent validity)

Questionnaire	Scale	FACIT-CD scale		
		Physical well-being	General perceptions	Emotional well-being
		r_s_ (95% CI)	r_s_ (95% CI)	r_s_ (95% CI)
SF-12	Physical function	0.20 (0.10–0.31)	NA	NA
	Physical role	0.18 (0.08–0.28)	NA	NA
	Bodily pain	0.16 (0.06–0.27)	NA	NA
	Emotional role	NA	NA	0.14 (0.04–0.25)
	Mental health	NA	NA	0.38 (0.29–0.47)
	General health	NA	0.32 (0.22–0.41)	NA
	Vitality	NA	0.28 (0.17–0.37)	NA
	Social role	NA	0.17 (0.06–0.27)	NA
	Physical component summary	0.17 (0.07–0.27)	NA	NA
	Mental component summary	NA	NA	0.34 (0.24–0.43)
EORTC QLQ-CX24	Sexual worry	−0.53 (−0.61 to −0.45)	NA	NA
	Sexual/vaginal function	−0.49 (−0.58 to −0.40)	NA	NA

r_s_ Spearman correlation coefficient, *CI* Confidence interval, *NA* Not available

Table 6 shows the sensitivity of the questionnaire to detect changes. The sensitivity of the relationship scale was considered low (ES = 0.17, SEM = 0.19). The sensitivities of the other scales that composed the FACIT-CD questionnaire were moderate (ES = 0.31–0.43; SEM = 0.29–0.52).

**Table 6:** Evaluation of the sensitivity of the FACIT-CD questionnaire

Scale	Pre-treatment (*n* = 179)		Post-treatment (*n* = 179)		Difference between means		p*	ES	SRM
	Mean	SD	Mean	SD	Mean	SD			
Physical well-being	23.1	4.3	24.9	4.5	1.7	4.6	< 0.001	0.40	0.37
Treatment satisfaction treatment	9.6	1.8	10.1	1.5	0.5	1.9	< 0.001	0.31	0.29
General perceptions	18.6	3.7	17.2	3.3	−1.4	2.7	< 0.001	−0.37	−0.51
Emotional well-being	30.3	6.8	33.2	5.6	2.9	5.5	< 0.001	0.43	0.52
Relationships	8.5	2.1	8.9	2.2	0.3	2.0	< 0.001	0.17	0.19
FACIT-CD	90.2	11.0	94.5	10.8	4.2	9.6	< 0.001	0.38	0.44

*SD* Standard deviation; p* = Wilcoxon; *ES* Effect Size, *SRM* Standardized response mean

The results of the responsiveness analysis indicated increase in the scores of the scales among women who reported improved health (4/5 scales) (Table 7). The magnitude of the change was moderate (ES = 0.27–0.58; SEM = 0.30–0.71). In this same group, the only scale in which the scores worsened after treatment was general perceptions (18.5–17.4; *p* = 0.001). The same scale indicated worsened HRQoL scores when the sensitivity of the FACIT-CD questionnaire was evaluated.

**Table 7:** Analysis of responsiveness of the FACIT-CD questionnaire

Scale	Health status	n	Pre-treatment	Post-treatment		Difference between means			ES	SRM	p*
			Mean	SD	Mean	SD	Mean	SD			
Physical well-being	No change	73	23.2	4.4	24.7	5.0	1.5	5.2	0.34	0.29	0.008
	Improvement	83	22.9	4.3	25.5	3.2	2.5	4.0	0.58	0.63	< 0.001
	Worsening	23	23.4	4.6	23.1	6.0	−0.3	4.5	−0.07	−0.07	0.87
Treatment satisfaction	No change	73	9.7	1.8	10.0	1.5	0.3	2.0	0.18	0.16	0.009
	Improvement	83	9.5	1.8	10.2	1.5	0.7	1.9	0.43	0.38	< 0.001
	Worsening	23	9.7	1.9	10.2	1.8	0.5	1.5	0.27	0.35	0.028
General perceptions	No change	73	18.5	4.0	16.6	3.8	−1.9	3.0	−0.48	−0.65	< 0.001
	Improvement	83	18.5	3.7	17.4	2.8	−1.0	2.6	−0.29	−0.41	0.001
	Worsening	23	19.1	2.7	18.3	2.5	−0.8	2.2	−0.29	−0.36	0.124
Emotional well-being	No change	73	30.3	6.6	33.3	5.2	3.0	5.5	0.45	0.54	< 0.001
	Improvement	83	29.8	7.3	32.9	6.2	3.1	5.7	0.42	0.54	< 0.001
	Worsening	23	32.0	5.3	34.0	4.7	1.9	5.3	0.36	0.36	0.038
Relationships	No change	73	8.6	2.1	8.8	2.2	0.2	1.9	0.009	0.10	0.021
	Improvement	83	8.4	2.2	9.0	2.0	0.6	2.0	0.27	0.30	0.003
	Worsening	23	9.0	2.1	9.1	2.9	0.1	2.1	0.06	0.06	0.284
FACIT-CD (total)	No change	73	90.4	11.2	93.6	11.7	3.1	10.7	0.27	0.29	0.003
	Improvement	83	89.2	10.9	95.2	9.9	5.9	8.4	0.55	0.71	< 0.001
	Worsening	23	93.4	10.3	94.9	11.3	1.4	9.7	0.14	0.15	0.429

*SD* Standard deviation, p* = Wilcoxon; *ES* Effect size, *SRM* Standardized response mean

Among women without changes in health between the assessments, the average scores remained unchanged (8.6–8.8; *p* = 0.021) and had low responsiveness (ES = 0.009; SEM = 0.10) only in the relationship scale (1/5 scales). In the other scales, the HRQoL scores improved with the exception of the general perception scale, which maintained the tendency of worsening after treatment.

Different results were found in the group of women who reported worsening of health between assessments. The decrease in the HRQoL scores was evident on the scales that assessed physical well-being and general perceptions (2/5 scale). There were no differences in the relationship scale and the total FACIT-CD score. However, the treatment satisfaction and emotional well-being scales improved.

## Discussion

To the best of our knowledge, this study is the first to validate a questionnaire (translated into Brazilian Portuguese) that measures the quality of life of women diagnosed with cervical intraepithelial neoplasia. The FACIT-CD questionnaire was developed by Rao et al. [6] in 2010. To date, no other studies have evaluated the psychometric properties of this instrument, which means that some comparisons are only exploratory.

The first test assessed the reliability of the questionnaire by analysing the internal consistency using Cronbach’s alpha coefficient. Results higher than 0.70 indicate that the items on the scales or domains are homogeneous or that they measure the same attribute. In this study, the value on the relationship scale was lower than expected (0.66). However, other authors support the hypothesis that Cronbach’s alpha values ​​higher than 0.60 could be acceptable [31]. Despite this assumption, we believe that a value of 0.70 ​​would be more desirable, and thus, we considered that the relationship scale did not achieve adequate internal consistency. Therefore, these results suggest that the relationship scale does not measure the same attribute because it addresses questions about the emotional support that women receive from their partner and family combined with questions about their relationships with friends and support in case of need [8]. We believe that further studies with other populations are necessary to compare the results and to determine whether the problems will be repeated.

The second stage of the study evaluated the reproducibility of the FACIT-CD questionnaire (i.e., the consistency of the results after repetition of the measurements). Most of the studies that assessed reproducibility used a period of 14 ± 5 days [32–34]. Despite this recommendation, the treatment of intraepithelial lesions is not related to sudden changes in health status. Therefore, the period between assessments used in this study was 30 days because this time frame represented the interval between the colposcopy examination and the second medical consultation. The lower limit of the 95% CI of the ICC on the treatment satisfaction scale was lower than 0.40, indicating low reproducibility (i.e., the variability in treatment satisfaction was greater than desired). Some factors reported by the study participants could justify this variability. The consultations were conducted by different physicians from the same team, which might lead to dissatisfaction or conversely a better evaluation in another consultation. The impact on the emotional factors of the patient might also influence this variable (e.g., whether the consultation was scheduled only to perform follow-up tests such as colposcopy or whether it was scheduled to inform the result of a test that would define a course of action). Emotional factors in these different instances (consultation for examination and consultation to receive laboratory test results) may explain this variability.

The best results were observed in the known-group validity analysis. The comparison of the groups of women with and without a diagnosis of CIN indicated significant differences in the scores on all scales. As expected, some scales showed worsening in the HRQoL scores in women without the disease. The reason for this difference was apparent in the items that composed the scales. In the scales that assessed treatment satisfaction and relationships, women without the disease responded “not at all” on various items, thereby decreasing the HRQoL scores as expected because they were not in treatment. The general perception scale evaluated items such as acceptance of infection and whether women could manage things that came up around the infection. A decrease in the HRQoL scores of women without the disease was expected for the items that composed the scale. These factors contributed to the decrease in the HRQoL scores in women without CIN compared with women with CIN based on the FACIT-CD total score. As expected, the scores of the other physical and emotional well-being scales were higher in women without the disease.

In an additional analysis, the test groups were classified based on the health status rating of each participant, with a lower score indicating a worse perception of the HRQoL. In this case, all scales showed significant differences. This analysis confirmed that the FACIT-CD questionnaire could differentiate the groups for which differences were expected.

The structural validity of the questionnaire was tested by confirmatory factor analysis. The results consistently confirmed the structure of the original questionnaire, which contained five factors. The only exception was in the fifth item of the physical well-being scale, which assessed the side effects of treatment. This item showed higher factor loading in the emotional well-being scale. The follow-up and treatment of women diagnosed with CIN have a greater emotional impact than physical impact [8]. Women who seek medical care after the diagnosis of changes in the Papanicolaou test rarely complain of physical changes but often complain of psychological changes [10–12]. This finding suggests that item GP5 ("I am bothered by side effects of treatment") is better allocated in the emotional well-being scale. On the other hand, confirmatory factor analysis is very sensitive to sample size, and its consistency requires a relatively large number of cases [35]. Therefore, an increase in the sample size may help confirm the new positioning of the variable in the model.

Regarding the convergent and divergent validities of the FACIT-CD questionnaire, we expected to find a correlation between the SF-12 and FACIT-CD questionnaire scales. However, no correlation was found, and the values were lower than 0.40. This result may have occurred because the SF-12 is a generic questionnaire that does not specifically address the questions explored in the FACIT-CD; therefore, the purposes of the evaluations were distinct. Another study that used a generic and a specific questionnaire reported the same problem when correlating the questionnaires [33]. This analysis was also conducted using the EORTC QLQ-CX24 questionnaire, which was developed to assess the HRQoL of women with cervical cancer and could easily calculate the scores of the scales and some items separately. Therefore, for this study, only the scales that assessed sexuality were used. The results of the correlation between the scales of the FACIT-CD and EORTC QLQ-CX24 questionnaires were satisfactory. In this case, it was possible to confirm the correlation of the FACIT-CD questionnaire with other dimensions for which a correlation was already expected.

Some of the women who participated in the first stage of the study and were treated surgically (LEEP) were interviewed again 6 months after surgery. In this analysis, improvements in the scale scores were expected after treatment using the SRM and relative efficiency (ES). The goal was achieved for all scales except for the general perception scale. The scale scores improved after surgery, and the sensitivity was considered low to moderate. The general perception scale indicated deterioration in the overall score; however, it was not possible to identify which items worsened. In the present study, we used the classical test theory (CTT), which tests the validity of an instrument (i.e., the ability to measure what it proposed to measure), for the psychometric analysis of the FACIT-CD questionnaire [36]. However, future studies should conduct other analyses using the item response theory (IRT) [37], which investigates items separately. [37] Furthermore, the responsiveness of the FACIT-CD questionnaire was evaluated using the same group in which sensitivity was measured before and after treatment. Other studies have used a methodology similar to ours to evaluate responsiveness [38–40]. However, in this case, the women were divided based on their self-reported health status. After treatment, the participants answered the FACIT-CD questionnaire and the first question of the SF-12 questionnaire (on health rating). Finally, the answers provided to this question before and after treatment were compared to allow the classification of the groups as improved, worsened, or no change in health status. In the group of 83 women who exhibited improved health, we noticed an increase in the scores of the scales, reflecting an improvement in HRQoL. The total score of the FACIT-CD indicated moderate responsiveness. Responsiveness was low in the groups of women who reported health worsening or had no changes in health status. The HRQoL scores improved even among women who reported not having good health. We believe that other health problems may have interfered with the responses and that there is no direct correlation between health worsening and the worsening of signs and symptoms resulting from CIN.

## Conclusions

Our results are encouraging and indicate that the FACIT-CD questionnaire is a promising tool for the analysis of HRQoL in women with CIN. Internal consistency and reproducibility were satisfactory. Regarding structural validity, only one item on the physical well-being scale was not kept in the original domain. The questionnaire was able to discriminate the groups according to disease status and self-rating of health. Sensitivity was low for the relationship scale, but moderate for the other scales. Responsiveness varied between the groups that denominate the self-perception of health as no change, improvement or worsening.

## Abbreviations

AGC-US: Atypical Glandular Cells not otherwise specified
ASC-H: Atypical squamous cells – cannot exclude HSIL
ASC-US: Atypical squamous cells of undetermined significance
CI: Confidence interval
CIN: Cervical intraepithelial neoplasia
CTT: Classical test theory
EORTC QLQ-CX24: The European Organization for Research and Treatment of Cancer Quality-of-Life questionnaire cervical cancer module
ES: Effect size
FACIT-CD: Functional Assessment of Chronic Illness Therapy – Cervical Dysplasia
HPV: Human papillomavirus
HRQoL: Health-related quality of life
HSIL: High grade squamous intraepithelial lesion
ICC: Intraclass correlation coefficient
IRT: Item response theory
LEEP: Loop electrosurgical excisional procedure
LSIL: Low grade squamous intraepithelial lesion
SF-12: Short-Form Health Survey
SRM: Standardized response mean
